# Whole-Genome Sequence of the First Extended-Spectrum β-Lactamase-Producing Strain of Salmonella enterica subsp. enterica Serovar Napoli

**DOI:** 10.1128/MRA.00973-18

**Published:** 2018-09-13

**Authors:** Mathieu Clément, Alban Ramette, Odette J. Bernasconi, Luigi Principe, Francesco Luzzaro, Andrea Endimiani

**Affiliations:** a Institute for Infectious Diseases, University of Bern, Bern, Switzerland; b Graduate School of Cellular and Biomedical Sciences, University of Bern, Bern, Switzerland; c Clinical Microbiology and Virology Unit, A. Manzoni Hospital, Lecco, Italy; University of Maryland School of Medicine

## Abstract

We report here the whole-genome sequence of the first extended-spectrum β-lactamase (ESBL)-producing strain of Salmonella enterica subsp. enterica serovar Napoli, LC0541/17, isolated from the stools of an ambulatory pediatric patient in northern Italy. The strain was of sequence type 474 (ST474) and possessed a 90-kb IncI1 ST49 plasmid carrying the *bla*_CTX-M-1_ ESBL gene.

## Abstract (precis)

We report here the whole-genome sequence of the first extended-spectrum β-lactamase (ESBL)-producing strain of Salmonella enterica subsp. enterica serovar Napoli, LC0541/17, isolated from the stools of an ambulatory pediatric patient in northern Italy.

## ANNOUNCEMENT

An increase in infections and outbreaks due to strains of Salmonella enterica subsp. enterica serovar Napoli has been recorded in Europe (1–3). This serovar has great propensity to cause bacteremia and is associated with hospitalization (4). However, only one draft genome sequence (GenBank accession number LFIH00000000) is available (5), which is from strain SN310 belonging to sequence type 474 (ST474) and is resistant to aminoglycosides and responsible for a multischool outbreak in Milan, Italy (1). To our knowledge, Salmonella Napoli strains resistant to third-generation cephalosporins have not yet been reported.

In June 2017, an ∼1-year-old girl living close to Milan was referred to the pediatrician because of diarrhea and abdominal pain. Salmonella Napoli strain LC0541/17 was isolated from the patient's stools. Susceptibility tests indicated that LC0541/17 was a suspected ESBL producer. Here, we describe the whole-genome sequence (WGS) of LC0541/17.

Genomic DNA was extracted from fresh overnight colonies grown on MacConkey agar at 36°C using the QIAamp DNA minikit (Qiagen). Both the MinION device (Oxford Nanopore Technologies) with the 1D library kit and the MiSeq platform (Illumina, Inc., done by GATC Biotech AG) with 2 × 150-bp reads were used to perform WGS. Adapters from the Nanopore and Illumina reads were removed using Porechop 0.2.3 (https://github.com/rrwick/Porechop) and Trimmomatic 0.33 (6), respectively. A total of 39,878 of the longest and best-quality Nanopore reads were extracted until reaching 500,001,524 bp (*N*_50_, 12,177 bp; maximum, 58,306 bp) using Filtlong 0.2.0 (https://github.com/rrwick/Filtlong), and contigs were assembled using Canu 1.5 (7). Contigs were circularized using Circlator 1.5.5 (8). The assembly was polished with Illumina-trimmed reads using Pilon 1.22, and annotation was performed using Prokka 1.13 (9, 10). Strain and plasmid STs were obtained using mlst 2.10 (https://github.com/tseemann/mlst), which relies on the PubMLST database (https://pubmlst.org/). Plasmid and antimicrobial resistance genes (ARGs) were found using ABRicate 0.8 (https://github.com/tseemann/abricate), which uses ResFinder and PlasmidFinder databases and pathogenicity islands using SPIFinder (https://cge.cbs.dtu.dk/services/SPIFinder/). Virulence gene profiles (VPs) were assessed using BLASTn. All software was run with default or advised parameters.

Two circular contigs were obtained, one chromosome and one plasmid (pLC0541_17). The chromosome size was 4,679,033 bp, with a GC content of 52.2%. Annotation indicated 4,555 coding sequences (CDSs) (not including ARGs), 22 rRNAs, 84 tRNAs, and 1 transfer-messenger RNA (tmRNA). LC0541/17 was of ST474, and five pathogenicity islands were detected, of which three were also observed in strain SN310. Ten Salmonella Napoli VPs were previously characterized (2). LC0541/17 was found to belong to the VP1 profile [*ssaQ mgtC spi_4D(siiD) sopB sopE1 bcfC*], which is the most prevalent (36%) among isolates from humans, animals, food, or the environment (2).

The size of pLC0541_17 was 90,558 bp, with a GC content of 49.1%. It was an IncI1 ST49 plasmid with 102 CDSs carrying the *bla*_CTX-M-1_ gene but no other ARGs. The CTX-M-1 group ESBLs are the most prevalent in Italy (11); however, only several 80- to 100-kb IncI1 ST49 CTX-M-1 plasmids have been reported in Denmark in Escherichia coli of either human, pig, or cattle origin (12). In particular, GenBank database comparison (BLASTn) revealed that pLC0541_17 was similar (99% identity, 99 to 100% coverage) to two of these plasmids (GenBank accession numbers KJ563250 and KM052220) and another from France (GenBank accession number LT985288). As for these three plasmids, pLC0541_17 carried the *bla*_CTX-M-1_ gene inserted into the following genetic environment: *pilJ orf477 bla*_CTX-M-1_ IS*EcpB1 pilI*.

The emergence of ESBL-producing Salmonella Napoli isolates is alarming. Public health institutions should coordinate surveillance programs to monitor the spread of these life-threatening pathogens.

### Data availability.

The sequences of the chromosome and plasmid are deposited in GenBank under accession numbers CP030838 and CP030839, respectively. Raw data are available in the Sequence Read Archive under accession number SRP156370.
